# The impact of developmental coordination disorder on educational achievement in secondary school

**DOI:** 10.1016/j.ridd.2017.10.014

**Published:** 2018-01

**Authors:** Ian Harrowell, Linda Hollén, Raghu Lingam, Alan Emond

**Affiliations:** a Centre for Child and Adolescent Health, School of Social and Community Medicine, Oakfield House, Oakfield Grove, University of Bristol, BS8 2BN, UK; b Institute of Health & Society, The Baddiley-Clark Building, Richardson Road, University of Newcastle, NE2 4AX, UK

**Keywords:** Developmental coordination disorder, Motor difficulties, Educational achievement, ALSPAC

## Abstract

**Background:**

Developmental coordination disorder (DCD) is a common developmental disorder but its long term impact on health and education are poorly understood.

**Aim:**

To assess the impact of DCD diagnosed at 7 years, and co-occurring developmental difficulties, on educational achievement at 16 years.

**Methods:**

A prospective cohort study using data from the Avon Longitudinal Study of Parents and Children (ALSPAC). National General Certificate of Secondary Education (GCSE) exam results and Special Educational Needs provision were compared for adolescents with DCD (n = 284) and controls (n = 5425).

**Results:**

Adolescents with DCD achieved a median of 2 GCSEs whilst controls achieved a median of 7 GCSEs. Compared to controls, adolescents with DCD were much less likely to achieve 5 or more GCSEs in secondary school (OR 0.27, 95% CI 0.21–0.34), even after adjustment for gender, socio-economic status and IQ (OR 0.6, 95% CI 0.44–0.81). Those with DCD were more likely to have persistent difficulties with reading, social communication and hyperactivity/inattention, which all affected educational achievement. Nearly 40% of adolescents with DCD were not in receipt of additional formal support during school.

**Conclusions:**

DCD has a significant impact on educational achievement and therefore life chances. Co-occurring problems with reading skills, social communication difficulties and hyperactivity/inattention are common and contribute to educational difficulties. Greater understanding of DCD among educational and medical professionals and policy makers is crucial to improve the support provided for these individuals.

## Highlights

• Adolescents with DCD in the UK performed poorly compared to controls in standardised national exams (GCSE) at 16 years.
• Co-occurring difficulties with reading, social communication and hyperactivity affected performance in those with DCD.
• Many of those with DCD were not receiving any additional formal educational support.

## What this paper adds?

Developmental coordination disorder (DCD) is one of the most common developmental conditions of childhood. However, its impact on longer term health and education is not well understood. This paper contributes robust epidemiological evidence of the persisting impact that DCD can have on learning and achievement in secondary school.

Using a population-based cohort, individuals with DSM-IV classified DCD at 7 years were 70% less likely to achieve 5 or more qualifications at 16 years than their peers. Co-occurrence of reading difficulties and other developmental traits, such as social communication difficulties and hyperactivity/inattention, were commonly present in adolescence and these difficulties contributed to poor educational achievement. This study also illustrates the extent to which DCD can be a hidden disability – 37% of those with the condition were not in receipt of additional formal teaching support.

These results demonstrate the impact DCD can have on educational attainment, and therefore on future life prospects. It is hoped this work will contribute to raising awareness and understanding of the impact of DCD and stimulate discussion about how best to support those with this complex condition.

## 1 Introduction

Developmental coordination disorder (DCD) is a common neurodevelopmental disorder characterised by deficits in both fine and gross motor coordination which have a significant impact on a child’s activities of daily living or school productivity (American Psychiatric Association, 2013). These deficits are present in the absence of severe intellectual or visual impairment, or another motor disability, such as cerebral palsy. It is thought to affect around 5% of school-aged children (American Psychiatric Association, 2013), but despite its high prevalence it remains one of the less well understood and recognised developmental conditions in both educational and medical settings.

Schoolwork of children with DCD often does not reflect their true abilities as they struggle with fine motor skills, including handwriting (Missiuna, Rivard, & Pollock, 2004). However, there is also evidence of a wider academic deficit involving reading, working memory and mathematical skills (Alloway, 2007, Dewey et al., 2002; Kaplan, Wilson, Dewey, & Crawford, 1998). Although initially identified on the basis of motor difficulties, the condition may develop into complex psychosocial problems, with difficulties in peer relationships and social participation (Sylvestre, Nadeau, Charron, Larose, & Lepage, 2013), bullying (Campbell, Missiuna, & Vaillancourt, 2012; Skinner and Piek, 2001, Wagner et al., 2012), low self-worth and perceived self-competence (Piek, Baynam, & Barrett, 2006), and internalising disorders, such as anxiety and low mood (Lingam et al., 2012). It may be that these sequelae lead to poor performance in school.

As well as secondary psychosocial consequences, those with DCD have a higher risk of displaying other developmental traits, such as hyperactivity and social communication difficulties, and specific learning disabilities, particularly dyslexia. (Kadesjo and Gillberg, 1999, Lingam et al., 2010, Sumner et al., 2016). Overlapping difficulties in two or more developmental and educational domains implies that discrete diagnosis of a single disorder is often not appropriate (Kaplan, Dewey, Crawford, & Wilson, 2001). In some individuals with DCD, it may be that co-occurring difficulties contribute to or explain some of the sequelae of the condition (Conti-Ramsden, Durkin, Simkin, & Knox, 2008; Loe & Feldman, 2007). Previous work has highlighted the importance of identifying co-occurring problems in DCD by demonstrating the mediating effects of social communication difficulties and hyperactivity can have on psychological outcomes (Harrowell, Hollén, Lingam, & Emond, 2017; Lingam et al., 2012). In the evaluation and management of a child with suspected DCD, consideration of other possible co-occurring difficulties is essential.

Although the body of literature on DCD demonstrates many reasons why a child with DCD might struggle in school (Zwicker, Harris, & Klassen, 2013), lack of awareness of the condition by medical and educational professionals is widespread, highlighted by parental reports of difficulty accessing support and services for their child (Missiuna, Moll, Law, King, & King, 2005; Novak, Lingam, Coad, & Emond, 2012). One study found that 43% of parents were not offered any practical support (Alonso Soriano, Hill, & Crane, 2015). Even when support is provided, it may not be appropriate for the child, which parents put down to lack of understanding of the condition (Maciver et al., 2011). A study of students in further and higher education found that those with dyslexia were more likely than students with DCD to receive Disability Student Allowance from the government, despite greater self-reported difficulties in the DCD group (Kirby, Sugden, Beveridge, Edwards, & Edwards, 2008). Furthermore, there were no differences between the types of support provided for these two different developmental disorders. This not only emphasises the poor recognition of DCD, but also lack of understanding of the specific needs of those with coordination problems.

By definition, children with DCD have motor difficulties that interfere with academic achievement (American Psychiatric Association, 2013). However, longitudinal studies of educational achievement in secondary school for those with DCD are few, and those that have been conducted lack strict diagnostic criteria or have been drawn from clinical samples. (Cantell, Smyth, & Ahonen, 1994; Gillberg and Gillberg, 1989, Losse et al., 1991). Thus, the primary aim of this research was to assess the impact of DCD on educational achievement in secondary school, using prospective data from a large population-based cohort study. Secondly, we aimed to assess the presence of co-occurring difficulties in reading ability, social communication problems and hyperactivity/inattention, and whether these impacted upon educational achievement in DCD. Thirdly, we aimed to determine how many of those meeting the criteria for DCD were identified for formal additional educational support in school, and assess whether provision of support was related to educational achievement.

## 2 Methods

### 2.1 Study participants

The Avon Longitudinal Study of Parents and Children (ALSPAC) is a population-based birth cohort which invited all pregnant women in the Avon area of southwest England, with expected dates of delivery between 1 April 1991 and 31 December 1992 to take part. The original sample comprised 14062 live-born children, with 13968 surviving to 1 year. ALSPAC has collected data on a large range of socio-economic, environmental and health measures for both parents and children; data were collected using questionnaires, face-to-face assessments and linked health and education data. Recruitment of participants and data collection have been described in detail elsewhere (Boyd et al., 2013). The study website contains details of all the data that are available through a fully searchable data dictionary (http://www.bris.ac.uk/alspac/researchers/data-access/data-dictionary/). Ethical approval for ALSPAC was obtained from the Local Research Ethics Committees, and this study was monitored by the ALSPAC Ethics and Law Committee.

### 2.2 Identification of developmental coordination disorder

Classification of children with DCD in this cohort and derivation of the measures used for the inclusion and exclusion criteria has been described in detail previously (Lingam, Hunt, Golding, Jongmans, & Emond, 2009). Children were defined as having DCD by applying the inclusion (Criteria A and B) and exclusion criteria (Criteria C and D) derived from the Diagnostic and Statistical Manual of Mental Disorders 4th Edition Text Revision (DSM-IV-TR) and adapted for research using the 2006 Leeds Consensus Statement (American Psychiatric Association, 2000; Sugden, Chambers, & Utley, 2006). Children were classified as having DCD at 7–8 years if they met all four criteria: (A) marked impairment of motor coordination, (B) motor coordination impairment significantly interferes with academic achievement or activities of daily living (ADL), (C) absence of another neurological/visual disorder, and (D) absence of severe learning difficulty (IQ > 70). DCD was defined in this cohort prior to the publication of the Diagnostic and Statistical Manual of Mental Disorders 5th Edition (DSM-V; American Psychiatric Association, 2013). However, the criteria for DCD were not significantly altered between the two editions, and so the criteria used in this cohort are compatible with the definition of DCD in the more recent DSM-V.

The ALSPAC coordination test, consisting of 3 subtests of the Movement Assessment Battery for Children Test (MABC; Henderson & Sugden, 1992), was applied by 19 trained examiners in a purpose-built research clinic at 7–8 years. Consistency was maintained by standardised training and ongoing supervision and training. The 3 subtests were chosen, following principal component analysis of the original standardization data set of the MABC, as they best represented the three domains of coordination: heel-to-toe walking (balance), placing pegs task (manual dexterity) and throwing a bean bag into a box (ball skills). Age-adjusted scores were derived by comparing the scores of each child to raw scores in the cohort itself. Those scoring under the 15th centile in the coordination test were considered to have a motor impairment, consistent with criterion A of the DSM-IV-TR definition (Schoemaker, Lingam, Jongmans, van Heuvelen, & Emond, 2013).

Academic achievement was assessed using linked educational data. As part of the national curriculum in the UK, literacy testing is undertaken at 7 years (Key Stage 1), which involves a writing test with questions on English grammar, punctuation and spelling. The writing test is scored 1–4 (4 being the best, 2 being the expected level at this age): those who scored 1 were considered to have significant difficulties with writing.

ADL were assessed using a parent-completed 23-item questionnaire at 6 years 9 months of age, containing items derived from the Schedule of Growing Skills II (Bellman, Lingam, & Aukett, 1996) and the Denver Developmental Screening Test II (Frankenburg & Dodds, 1967). The questions represented skills the child would have been expected to achieve by 81 months. It assessed for difficulties in developmentally age-appropriate skills with which children with DCD struggle, such as self-care, playing, and gross and fine motor skills. Age-adjusted scores were calculated by stratifying the child’s age and those scoring below the 10th centile were considered to have significant impairment.

IQ was measured at 8.5 years by trained psychologists in a research clinic using a validated and shortened form of the Wechsler Intelligence Scale for Children-III (WISC-III; Wechsler, Golombok, & Rust, 1992; Connery, Katz, Kaufman, & Kaufman, 1996). Alternate items were used for all subtests, with the exception of the coding subtest which was administered in its full form. Using the look-up tables provided in the WISC-III manual, age-scaled scores were obtained from the raw scores and total scores were calculated for the Performance and Verbal scales. Prorating was performed in accordance with WISC-III instructions. IQ was assumed to be stable over time (Schneider, Niklas, & Schmiedeler, 2014). Those with an IQ < 70 and those with known visual, developmental or neurological conditions were excluded from case definition of DCD (criterion C and D).

Children who scored below the 15th centile on the ALSPAC coordination test (criterion A), and had significant difficulties with writing in their Key Stage 1 handwriting test or were below the 10th centile on the ADL scale (criterion B), were defined as having DCD. At 7–8 years, a cohort of 6902 children had all the data required for full assessment, and 329 children met the criteria for DCD.

### 2.3 Educational achievement

ALSPAC obtained linked educational data from the National Pupil Database, a central repository for pupil-level educational data, and from the Pupil Level Annual School Census, which captures pupil-level demographic data about special needs support. The linked educational data in ALSPAC covers pupils in England and in state-funded schools; therefore pupils from Wales or in independent schools are excluded from this analysis.

Academic achievement at 16 years was assessed using the results from the General Certificate of Secondary Education (GCSE) at Key Stage 4. These are the national achievement exams, covering a range of subjects (English and Maths are mandatory), undertaken at the end of compulsory schooling by all children in state schools in England. They are marked by anonymous external examiners and given grades ranging from A*-G. For the purpose of our analysis, we dichotomised the cohort into those who did and did not achieve 5 or more GCSEs graded A*-C, which is a widely used marker of performance in the UK.

Special Educational Needs (SEN) provision data were obtained at 9–10 years (year 5, end of primary school) and 11–12 years (year 7, start of secondary school). At 9–10 years old, SEN status was divided into six groups. ‘No SEN provision’ indicated no formal support was being provided. ‘SEN provision without statement levels 1–4’ indicated extra support was being provided, with increasing level indicating increasing level of severity. ‘Statement of SEN’ indicated that a statutory assessment by the Local Authority had been undertaken and a statement of extra support required had been created. SEN status at 11–12 years was categorised into four main groups: ‘no SEN provision’, ‘School action’ (which indicated the child had been recognised as not progressing satisfactorily and extra support was being provided internally), ‘School action plus’ (which indicated the child had not made adequate progress on ‘school action’ and the school had sought external help from the Local Authority, the National Health Service or Social Services), and ‘Statement of SEN’. For the purpose of our analyses, at both time points, SEN status was dichotomised: those receiving no formal support at all and those receiving some level of support.

### 2.4 Confounding variables

Gender, gestation, birthweight, socioeconomic status and IQ are known to be associated with DCD (Lingam et al., 2009), and have well-established links with academic performance. They were therefore selected as confounders for the multi-variable analysis.

Gender, gestation and birthweight were extracted from the birth records in ALSPAC. Family adversity was measured using the ALSPAC Family Adversity Index (FAI). This is derived from responses to a questionnaire about childhood adversity and socio-economic status which mothers completed during pregnancy. The index comprises 18 items which are assigned a score of 1 if adversity is present and 0 if it is absent, giving a total possible score of 18. The FAI includes the following factors: age at first pregnancy, housing adequacy, basic amenities at home, mother’s educational achievement, financial difficulties, partner relationship status, partner aggression, family size, child in care/on risk register, social network, known maternal psychopathology, substances abuse and crime/convictions.

### 2.5 Co-occurring difficulties

Reading ability (Lingam et al., 2010), social communication difficulties (Conti-Ramsden et al., 2008) and hyperactivity/inattention (Loe & Feldman, 2007) were selected as important conditions to be adjusted for in the multi-variable analysis, as they often co-occur with DCD and can impact on educational achievement.

Reading ability was measured in research clinics at 13.5 years using the Test of Word Reading Efficiency (TOWRE), a short test which measures an individual's ability to pronounce printed words and phonemically regular non-words accurately and fluently. Words are used to assess sight word reading efficiency and non-words to assess decoding efficiency. The scoring is based on the number of words/non-words read quickly and correctly during 45 s (Torgesen, Wagner, & Rashotte, 1999).

Social communication difficulties were measured at 16.5 years using the Social and Communication Disorders Checklist (SCDC; Skuse, Mandy, & Scourfield, 2005), completed by the main caregiver. It comprises 12 items relating to the child’s social communication ability and cognition, each with 3 responses (not true, quite or sometimes true/very or often true, scoring 0/1/2 respectively). Answers are summed to give a score between 0 and 36. A score of 9 or above was used to predict social communication difficulty trait (Humphreys et al., 2013).

Hyperactivity/inattention was measured at 15.5 years using the self-reported Strengths and Difficulties Questionnaire hyperactivity-inattention subscale (SDQ; Goodman & Goodman, 2009). This subscale of the questionnaire comprises 5 items relating to behaviours indicating hyperactivity, each with 3 responses (not true/somewhat true/certainly true, scored 0/1/2 respectively). A score in the top decile of the ALSPAC cohort was taken as indicating significant hyperactivity (Goodman, 2001).

### 2.6 Statistical analyses

The two-sample test for proportions, Student’s *t*-test and the Mann-Whitney *U* test were used, where appropriate, to assess differences between groups. Logistic regressions were used to assess the impact of DCD on educational achievement. Multi-variable models were created to adjust for the effect of the confounding variables and mediating variables sequentially. Covariates significant at the 5% level in the univariate analyses were included in the multivariable models. Model 1 adjusted for gender, birthweight, gestation and socioeconomic status. Model 2 adjusted for IQ. Model 3 adjusted for reading ability. Model 4 adjusted for the developmental traits of social communication difficulties and hyperactivity/inattention. Pearson goodness of fit chi-squared tests were used to ensure satisfactory model fit.

Multiple imputation using chained equations (ICE) was used to impute missing data in the covariates only (Appendix A in Supplementary material). Analysis of imputed datasets helps to minimise attrition bias and improve precision of estimates (Sterne et al., 2009). Logistic regressions were used to determine which variables strongly predicted missingness and these were included in the ICE prediction models. Twenty imputations were performed. All analyses were performed using Stata v. 14.1 (StataCorp, College Station, TX, USA). An alpha level of 0.05 was used for all statistical tests.

## 3 Results

### 3.1 Sample characteristics

Of those previously assessed for DCD at age 7 years, educational data at 16 years were available for 5709 adolescents, including 284 (4.9%) of those who met the criteria for DCD. These represented 86% (284/329) of those originally diagnosed with DCD. Characteristics of those with and without educational outcome data available are shown in Appendix B in Supplementary material.

Characteristics of the adolescents with DCD and controls with educational data available are compared in Table 1. In this follow-up cohort, when compared to controls, those with DCD were more likely to be male, have been born prematurely, have a low birth weight, have a lower IQ and have experienced greater family adversity.

**Table 1:** Characteristics of the cohort, comparing controls to those with DCD.

	Control (max. n = 5425)	DCD (max. n = 284)	p
Gender – male, n (%).	2676 (49) n = 5425	175 (62) n = 284	z = 4⋅27,<0.001^a^
Gestation – <37 weeks, n (%)	289 (5) n = 5425	27 (10) n = 284	z = 3.68, <0.01^a^
Birthweight – <2500 g, n (%)	237 (4) n = 5369	28 (10) n = 279	z = 4.82, <0.001^a^
Family adversity – worst quartile, n (%)	712 (13) n = 5425	54 (19) n = 284	z = 2.90, <0.01^a^
IQ at 8 years – mean (standard error)	105.4 (0.21) n = 4504	93.5 (1.08) n = 202	t(4704) = 10.81, <0.001^b^

a Two-sample test for proportions.

b Student’s *t*-test.

### 3.2 Academic achievement and SEN status

There were substantial differences in educational achievement and SEN status between the controls and DCD group (Table 2). The control group achieved a median of 7

**Table 2:** Educational achievement at Key Stage 4 and SEN provision, comparing controls to those with DCD.

		Control (max. n = 5425)	DCD (max. n = 284)	p
Total number of GCSEs graded A*-C achieved: median (IQR)^a^	*Whole group*	7 (4–9) n = 5425	2 (0–8) n = 284	z = 10.50, p < 0.001^b^
*Female*		8 (5–9) n = 2749	3 (0–8) n = 109	z = 6.45, p < 0.001^b^
*Male*		7 (3–9) n = 2676	2 (0–7) n = 175	z = 7.73, p < 0.001^b^

Proportion of adolescents achieving 5 or more GCSEs graded A*-C: n (%)^a^	*Whole group*	3822 (71) n = 5425	111 (39) n = 284	z = 11.41, p < 0.001^c^
*Female*		2079 (76) n = 2749	50 (46) n = 109	z = 7.08, p < 0.001^c^
*Male*		1743 (65) n = 2676	61 (35) n = 175	z = 7.97, p < 0.001^c^

Special Educational Needs Provision – n (%)				
*No special provision at any point*		4270 (83) n = 5165	100 (37) n = 273	z = 18.77, p < 0.001^c^
*SEN provision only in primary school*		372 (7)	42 (15)	z = −4.92, p < 0.001^c^
*SEN provision in only secondary school*		166 (3)	25 (9)	z = −5.41, p < 0.001^c^
*SEN provision in both primary and secondary school*		357 (7)	106 (39)	z = −18.37, p < 0.001^c^

a Educational achievement outcomes have been presented for the groups as a whole and also stratified by gender.

b Mann-Whitney *U* test.

c Two-sample test ofproportions. IQR – Inter-quartile range.

**Fig. 1:**
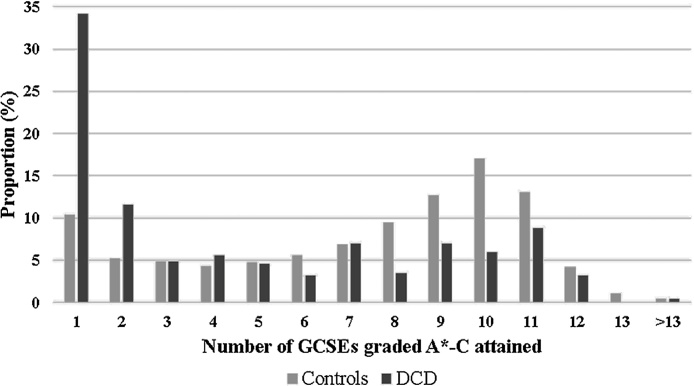
Number of GCSEs graded A*-C achieved by controls and those with developmental coordination disorder (DCD).

GCSEs graded A*-C, with 70% overall achieving 5 or more GCSEs at this level. In contrast, those with DCD achieved a median of 2 GCSEs graded A*-C, with 39% overall achieving 5 or more GCSEs. Fig. 1 illustrates the skewed distribution of GCSE attainment in the DCD group.

When results were stratified by gender, in the control group girls performed significantly better than boys in achieving 5 or more GCSEs graded A*-C (76% vs. 65%, z = 8.89, p < 0.001). In the DCD group, girls were also more likely than boys to achieve 5 or more GCSEs graded A*-C, but this association did not reach significance (46% vs. 35%, z = 1.84, p = 0.064).

The majority of those in the control group were not receiving any SEN provision at both time points; 895/5165 (17%) of controls received some form of SEN provision during their school career. In contrast, of those in the DCD group, 175/273 (63%) received some form of SEN support (z = −18.77, p < 0.001). In the DCD group, 15% were in receipt of support in primary school only and 9% in secondary school only, with 39% being recognised as needing extra support in both primary and secondary school.

A sub-group analysis was performed to compare educational achievement for those who received SEN support and those who did not (Table 3). Differences between the groups were assessed using the two-sample test for proportions. Controls who received no SEN support were no more likely to achieve 5 or more GCSEs graded A*-C than those with DCD who received no SEN support (76% vs. 70%, z = 1.37, p = 0.17). Controls who received SEN support at any time were more likely to achieve 5 or more GCSEs graded A*-C than those with DCD who received SEN support at any time (27% vs. 16%, z = 3.00, p < 0.01). In the control group, those who received no SEN support were more likely to achieve 5 or more GCSEs graded A*-C than those who received SEN support at any time (76% vs. 27%, z = 28.10, p < 0.001). This was similar to the DCD group: those who received no SEN support were more likely to achieve 5 or more GCSEs graded A*-C than those who received SEN support at any time (70% vs. 16%, z = 6.85, p < 0.001).

**Table 3:** Educational achievement of those receiving Special Educational Needs (SEN) provision compared to those without.

		No SEN provision	SEN provision in primary school	SEN provision in secondary school	SEN provision in both primary and secondary school
Controls. *(total n* *=* *5069)*	Proportion achieving 5 or more GCSEs graded A*-C, n (%)	3202 (76) n = 4200	137 (38) n = 363	65 (40) n = 161	31 (9) n = 345
	Number of GCSEs graded A*-C: median (IQR)	8 (5–9)	3 (1–6)	3 (0–7)	1 (0–5)

DCD *(total n* *=* *267)*	Proportion achieving 5 or more GCSEs graded A*-C, n (%)	68 (70) n = 98	11 (27) n = 41	6 (24) n = 25	10 (10) n = 103
	Number of GCSEs graded A*-C: median (IQR)	8 (3–10)	1 (0–5)	0 (0–4)	0 (0–2)

IQR – Inter-quartile range.

### 3.3 Co-occurring difficulties

Table 4 details the co-occurring difficulties reported for the control and DCD groups. Those with DCD were more likely to have difficulty with reading on both the word and non-word tests, social communication difficulties and hyperactive-inattentive behaviours.

**\Table 4:** Co-occurring difficulties in those with DCD compared to controls.

	Control (max. n = 5425)	DCD (max. n = 284)	p
Sight word efficiency – TOWRE word score: median (IQR).	84 (77–90) n = 3907	77.5 (69.5–85) n = 184	z = 7.60, p < 0.001^a^
Decoding efficiency – TOWRE non-word score: median (IQR)	53 (46–58) n = 3897	47.5 (39–56) n = 182	z = 5.30, p < 0.001^a^
Social communication difficulties – SCDC score > 8: n (%)	354 (12) n = 3084	31 (22) n = 140	z = −3.51, p < 0.001^b^
Hyperactivity – worst decile on SDQ subscale: n (%)	349 (9) n = 3781	30 (18) n = 169	z = −3.93, p < 0.001^b^

^a^Mann-Whitney U test, ^b^Two-sample test for proportions. IQR – Inter-quartile rang; SCDC – Social and Communication Disorders Checklist; SDQ – Strength andDifficulties Questionnaire; TOWRE – Test of Word Reading Efficiency.

### 3.4 Multi-variable logistic regression

**Fig. 2:**
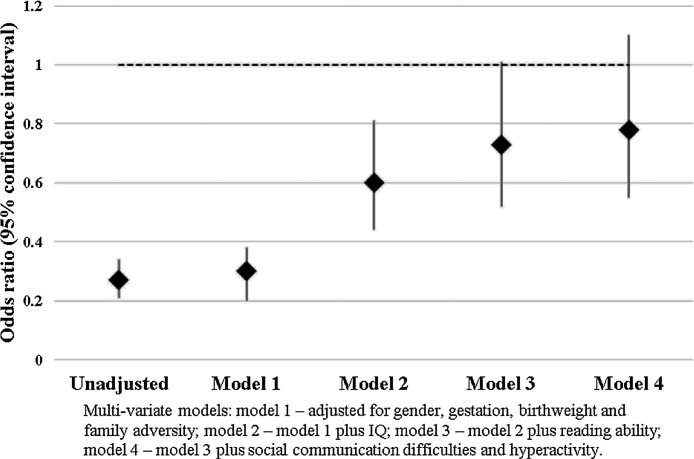
Odds ratios for achieving 5 or more GCSEs graded A*-C for those with DCD compared to controls, using multiple imputation data.

The results of the multi-variable logistic regressions, using the multiple imputation dataset, are shown in Fig. 2. Results of the analyses of all available data are presented in Appendix C in Supplementary material. In the unadjusted model, compared to their peers, individuals with DCD were much less likely to achieve 5 or more GCSEs graded A*-C (Odds Ratio [OR] 0.27, 95% Confidence Interval [CI] 0.21–0.34). Even after adjusting for confounding variables and IQ, they were still significantly less likely to achieve 5 or more GCSEs (OR 0.60, 95%CI 0.44–0.81). After adjusting for reading ability, this association was attenuated (OR 0.73 95%CI 0.52–1.01) and was attenuated further after adjustment for poor social communication skills and hyperactivity/inattention (OR 0.78, 95% CI 0.55–1.10).

## 4 Discussion

### 4.1 Summary of results

This prospective study using a large population-based UK cohort and strict diagnostic criteria has shown that children with DCD are much less likely to achieve 5 or more GCSEs graded A*-C in secondary school when compared to their peers. Children with DCD were more likely to have difficulties with reading, social communication and hyperactivity/inattention, and these problems contributed to poor achievement in school. Those with DCD were more likely to be identified as requiring formal SEN support than controls, although over a third of those with the condition were not identified as requiring formal SEN support. Adolescents with DCD without SEN support did not perform significantly worse in their GCSEs than controls without SEN support. Conversely, adolescents with DCD who were receiving SEN support performed worse in their exams than controls with SEN support.

### 4.2 Discussion of results

The proportion of pupils achieving 5 or more GCSE qualifications at grades A*-C is not only used as an attainment indicator in the UK (Department for Education, 2016), but at an individual level it is often specified as a requirement to obtain places in further post-16 school education, in college or in certain jobs and apprenticeships. Poor performance in GCSEs can therefore have a large negative impact on an individual’s life prospects, depending on what they wish to do, as it may limit options for the next steps in their educational or vocational development. It is known that median hourly pay rate increases incrementally with increasing level of qualification, and that those with a higher level of qualification are more likely to be in skilled and better paid jobs (Office for National Statistics, 2011). Therefore, those with DCD are at a considerable disadvantage not only educationally, but also economically in their future careers. DCD is one of the more prevalent but less well-understood developmental conditions, and so this impact on life course is concerning.

As well as accounting for the effects of important confounders and IQ on the relationship between DCD and educational achievement, we assessed the impact of co-occurring difficulties with reading, social communication and hyperactivity/inattention. As the effect of these traits were consecutively adjusted for in the multi-variable model, the difference between the control and DCD group was attenuated, which is reflected in the sequentially attenuated odds ratios from model 2 to model 4. This suggests that co-occurring developmental traits, which are common in DCD (Lingam et al., 2009), may account for some of the under-achievement at GCSE level in those with DCD compared to controls. In the fully adjusted model, those with DCD were 22% less likely to achieve 5 or more GCSEs compared to controls. However, it should be noted that the confidence intervals are substantially wider in models 3 and 4, which likely reflects the greater attrition in response rates for the variables in these models. These results emphasise the need for a holistic view of a child with DCD – problems with school work may not just be solely due to motor deficits and they should not be viewed as ‘just clumsy’. Co-occurring difficulties in other developmental domains should be actively sought, and if present, addressed in any support provided.

Of those who met the criteria for DCD, 37% did not receive any formal SEN provision. This corroborates what has been reported in qualitative work with parents (Missiuna et al., 2005) and illustrates how DCD is often a hidden disability, as motor deficits can be subtle and learning impairments difficult to recognise. This is important to realise because if missed, children with DCD may start to avoid situations which draw attention to their poor coordination as they become more aware of their motor difficulties, to try to minimise embarrassment or bullying. Previous work on this cohort has shown that adolescents with DCD report more bullying and less supportive friendships than their peers (Harrowell et al., 2017). This, along with reducing engagement at school, will contribute to poor perceived efficacy and low self-esteem (Skinner & Piek, 2001). The combined impact of these secondary problems, as well as the primary motor deficit, will ultimately compound poor performance in school. Poor academic performance may further exacerbate engagement at school, leading to a complex vicious circle.

Within the DCD group, those with formal SEN provision performed significantly worse in their exams than their counter-parts without SEN provision. The explanation for this is likely two-fold. Firstly, those with the most severe motor deficits, who will have most difficulty in school, are likely to be recognised as requiring extra help. Secondly, it may be that those receiving SEN support were those with co-occurring difficulties, whose multiple problems compound their poor performance, and are more likely to be recognised as needing support. A previous study which found a similar pattern in children with specific language impairments; those who had a ‘Statement of SEN’ performed worse than those without one in their secondary school exams (Durkin, Simkin, Knox, & Conti-Ramsden, 2009).

### 4.3 Implications of the findings

Guidelines for diagnosis, assessment and intervention in DCD were published in 2012 based on the best available evidence (Blank, Smits-Engelsman, Polatajko, & Wilson, 2012), and were more recently adapted for the UK context following a multi-disciplinary consultation (Barnett, Hill, Kirby, & Sugden, 2015). The evidence provided by this study supports the need to raise awareness of the condition and its consequences not only among educational and clinical health professionals, but also policy makers. Clearly defined pathways for children with DCD are required to coordinate services appropriately. The work of organisations like the Dyspraxia Foundation (Dyspraxia Foundation, 2016) and Movement Matters (Movement Matters UK, 2016) provide information about DCD to families, carers, teachers and clinicians, with the aim of improving awareness of and support for individuals with condition.

Since March 2014, policy for SEN provision in the UK has been reformed, with the ‘Statement of SEN’ being replaced by an incorporated ‘Educational, Health and Care Plan’ in the Children and Families Act 2014 (Department for Education, 2014). How this will impact upon care of those with DCD remains to be seen, and implementation is likely to vary between regions. It is hoped that the results of the work on DCD in ALSPAC will contribute to improving awareness and understanding of DCD and subsequently improved planning of support.

Increased awareness and understanding of the condition needs to be followed by improved intervention. The evidence base for what interventions work best in DCD is limited. Interventions which are task-orientated and individualised appear to have the largest effect on motor skills, based on a recent systematic review of high quality randomised controlled trials (Preston et al., 2016). As highlighted in this study, however, motor skills may only be one domain that requires attention in a child identified as having DCD. More research is needed on the benefit of interventions aimed at addressing other developmental domains concurrently, such as social communication skills (Piek et al., 2015). Further, there is a paucity of research into the needs of adolescents and adults with DCD. Many young people with DCD continue to experience motor and psychosocial difficulties after they have finished school and it is important that they are not forgotten and are supported to gain employment (Kirby, Edwards, & Sugden, 2011; Kirby, Williams, Thomas, & Hill, 2013).

### 4.4 Strengths and weaknesses of the study

The key strength of this study is the use of a large, prospective, population-based sample which is broadly representative of the UK (Boyd et al., 2013), and avoids the biases introduced in clinical samples. Due to the large amount of data collected in ALSPAC, we were able to strictly define our cases of DCD according to DSM-IV-TR criteria, and also to assess the impact of important co-occurring problems on our main outcome. The use of linked educational data was also a strength of the study.

The main weakness of this study, as with all prospective cohorts, is attrition. Through the use of linked education data, we were able to minimise loss-to-follow-up in our primary outcome measure. For those who had GCSE data available (n = 5709), 81% had all the covariates available for multi-variable models 1 and 2. Those who did not have GCSE data available at 16 years were more likely to have higher IQ and a higher level of maternal education (Appendix A in Supplementary material), a pattern which was seen in both the control and DCD groups and so should not systematically affect our results and thus our conclusions. The largest attrition occurred in model 4 (social communication deficits and hyperactivity), which relied on questionnaires filled out between 14 and 16 years. In ALSPAC, it is recognised that those who are lost to follow-up in clinics and questionnaires tend to come from lower socio-economic backgrounds, have lower IQ and are more likely to be male (Boyd et al., 2013). These are factors which are also associated with DCD and so differences between the groups may be under-estimated. We have attempted to minimise any bias introduced by missing data in the covariates by the use of multiple imputation, a well-validated statistical technique (Sterne et al., 2009).

Another drawback is that no measure of motor competence was performed in adolescence. Motor skill difficulties identified in childhood do not always persist into adolescence (Cantell et al., 1994) and so there may be some in the DCD group who no longer have significant motor deficits. Furthermore, we were only able to comment on whether the child had been identified as requiring formal SEN provision, but not the level or type of support, or whether intervention had any impact on the core deficits in DCD.

## 5 Conclusions

DCD is an important but poorly understood developmental condition that has a stark impact upon educational achievement at the end of secondary schooling, which will affect an individual’s future prospects. Co-occurring developmental conditions are common, and may contribute to poor educational outcomes. Addressing these co-occurring difficulties, as well as motor deficits, is necessary to improve outcomes. Increased awareness and understanding of the condition amongst healthcare and educational professionals and policy makers is vital to improve the support provided for those with DCD.

## Conflict of interests

The authors declare that they have no conflict of interests relating to this article.

## Funding

This research did not receive any specific grant from funding agencies in the public, commercial, or not-for-profit sectors. The UK Medical Research Council and the Wellcome Trust (Grant ref: 102215/Z/13/Z) and the University of Bristol provide core support for ALSPAC.
