# Supplementary material for: The impact of developmental coordination disorder on educational achievement in secondary school
Source: Res Dev Disabil. 2018 Jan;72:13–22. doi: 10.1016/j.ridd.2017.10.014 (PMC5770330; doi:10.1016/j.ridd.2017.10.014)
Supplement: Supplementary file 1 [file mmc1.docx]

**Appendix A** Missing data and multiple imputation

Missing data are a problem in all cohort studies, particularly when data are collected over a long period and from different sources, such as in the Avon Longitudinal Study of Parents and Children. Of the 6902 children assessed for DCD at 7 years, 5709 had GCSE data available at 16 years.

The multi-variate models for logistic regression used 8 different confounders/covariates. There were no missing data in the gender, gestation and family adversity variables. Of the remaining covariates, 2369 children (41.5%) had no missing values and 839 children (14.7%) had a single missing value, with 2501 (43.8%) having between 2-5 missing values in the fully adjusted model. The variables with most missing data were social communication difficulties and hyperactivity.

Multiple imputation is a technique which can help improve validity of research with significant missing data by accounting for bias introduced by loss to follow-up (Sterne et al. 2009). In this study, multiple imputation using chained equations was performed using the “ICE” command in Stata v.14.1 (StataCorp, College Station, TX, USA). Twenty stacked datasets were generated and used in the final analysis which used the “MIM” command.

Variables used in the imputation model were all of those included in the final regression model and those which predicted missingness in the covariates only. In ALSPAC, it is known that those from lower socio-economic groups are less likely to be followed up. Therefore, we used logistic regression to explore factors were associated with missingness in our sample. The factors which most strongly predicted missingness (p<0.05) were gender, highest maternal education and IQ; these were all included in the final imputation model. Binary and categorical variables used logistic, ordinal and multinomial as appropriate, specified in the imputation model. IQ was normally distributed and used linear regression.

By including extra variables which explain missingness in the imputation model, as well as the covariates of interest, the assumption of “missing at random” is supported.

| **Appendix B**  Comparison of the characteristics of those with and without educational data available at 16 years, for the control and DCD groups separately. | | | | | | |
| --- | --- | --- | --- | --- | --- | --- |
|  | **Controls** | |  | **DCD** | |  |
|  | Responders  (max. n=5425) | Non-responders (max. n=892) | p | Responders  (max. n=284) | Non-responders  (max. n=45) | p |
| Gender – male: n (%) | 2676 (49.3) n=5425 | 473 (53.1) n=892 | **0.041^a^** | 175 (61.6) n=284 | 32 (71.1) n=45 | 0.221^a^ |
| Gestation - <37 weeks: n (%) | 289 (5.3) n=5425 | 45 (5.0) n=892 | 0.727^a^ | 27 (9.51) n=284 | 3 (6.7) n=45 | 0.539^a^ |
| Birthweight - <2500g: n (%) | 237 (4.4) n=5369 | 46 (5.2) n=884 | 0.295 ^a^ | 28 (10.0) n=279 | 2 (4.4) n=45 | 0.230 ^a^ |
| Family Adversity Index – worst quartile: n (%) | 712 (13.1) n=5425 | 105 (11.8) n=892 | 0.264^a^ | 54 (19.0) n=284 | 10 (22.2) n=45 | 0.613^a^ |
| Highest maternal education – GCSE level: n (%) | 4512 (86.5) n=5219 | 626 (72.0) n=869 | **<0.001^a^** | 233 (87.3) n=267 | 33 (76.7) n=43 | 0.067^a^ |
| IQ at 8 years - mean (standard error) | 105.4 (0.23) n=4504 | 108.5 (0.61) n=716 | **<0.001^b^** | 93.5 (1.12) n=202 | 100.9 (3.32) n=31 | **0.010^b^** |
| *^a^Two-sample test for proportions , ^b^Students t-test.* | | | | | | |

| **Appendix C**  Odds ratios for achieving 5 or more GCSEs at grades A*-C for those with DCD compared to controls, using all available data. | | | | |
| --- | --- | --- | --- | --- |
|  | Odds Ratio | 95% Confidence Interval | p | n |
| Unadjusted | 0.27 | 0.21 – 0.34 | **<0.001** | 5709 |
| Model 1 | 0.30 | 0.23 – 0.39 | **<0.001** | 5648 |
| Model 2 | 0.68 | 0.48 – 0.95 | **0.033** | 4651 |
| Model 3 | 0.79 | 0.51 – 1.22 | 0.290 | 3257 |
| Model 4 | 1.01 | 0.6 – 1.73 | 0.940 | 2369 |
| *Multi-variate models: model 1 – adjusted for gender, gestation, birthweight and family adversity; model 2 – model 1 plus IQ; model 3 – model 2 plus reading ability; model 4 – model 3 plus social communication difficulties and hyperactivity/inattention.* | | | | |
